# Wogonin Suppresses Melanoma Cell B16-F10 Invasion and Migration by Inhibiting Ras-Medicated Pathways

**DOI:** 10.1371/journal.pone.0106458

**Published:** 2014-09-09

**Authors:** Kai Zhao, Libin Wei, Hui Hui, Qinsheng Dai, Qi-Dong You, Qing-Long Guo, Na Lu

**Affiliations:** State Key Laboratory of Natural Medicines, Jiangsu Key Laboratory of Carcinogenesis and Intervention, Key Laboratory of Drug Quality Control and Pharmacovigilance, Ministry of Education, China Pharmaceutical University, Nanjing, People's Republic of China; Duke University Medical Center, United States of America

## Abstract

The patients diagnosed with melanoma have a bad prognosis for early regional invasion and distant metastases. Wogonin (5,7-dihydroxy-8-methoxyflavone) is one of the active components of flavonoids that extracts from *Scutellariae radix*. Several previous studies reported that wogonin possesses antitumor effect against leukemia, gastrointestinal cancer and breast cancer. In this study, we used melanoma cell B16-F10 to further investigate the anti-invasive and anti-migratory activity of wogonin. Our date showed that wogonin caused suppression of cell migration, adhesion, invasion and actin remodeling by inhibiting the expression of matrix metalloproteinase-2 and Rac1 *in vitro*. Wogonin also reduced the number of the tumor nodules on the whole surface of the lung *in vivo*. Furthermore, the examination of mechanism revealed that wogonin inhibited Extracellular Regulated protein Kinases and Protein Kinase B pathways, which are both medicated by Ras. Insulin-like growth factor-1-induced or tumor necrosis factor-α-induced invasion was also inhibited by wogonin. Therefore, the inhibitory mechanism of melanoma cell invasion by wogonin might be elucidated.

## Introduction

In skin cancer, metastatic melanoma is the most fatal one. It is characterized by a bad prognosis for aggressive invasion and high drug resistance [1]. Invasion gives melanoma cells the ability to break out of tissue compartments and spread locally. Invasive cells can remodel cytoskeleton and structure of the extracellular matrix (ECM) to move through tissue barriers, which includes adhesion, invasion and migration of cancer cells [2]. Rac1, which is Ras-related protein, plays key roles in cytoskeletal reorganization, transcriptional regulation and membrane trafficking [3]. Matrix metalloproteinases (MMPs), a family of well-characterized structural related zinc-dependent proteases, plays a crucial role in ECM degradation [4], [5]. MMP-2 and MMP-9 are often over-expressed in malignant cancer. Specifically, MMP-2 is a better prognostic indicator in melanoma [6]. Although broad spectrum MMP inhibitors showed weak potential in tumor therapy, MMPs as key markers for prognosis and regulatory factor accord with the clinical standpoint as for melanoma.

The Ras proteins (H-Ras, K-Ras and N-Ras) are GTPases that regulate various cellular activities, including migration and cytoskeletal dynamism. Approximately 30% of tumors have activating mutations in Ras isoforms, and malignant melanomas mainly have activating mutations in N-Ras [7]. It results in uncontrolled activity of Ras proteins and continuous stimulation of Ras downstream signaling, specifically the ERK and PI3K-AKT pathways [8]. In addition, enhanced Ras signaling frequently accompanies with consequential NF-κB activation in cancer [9]. The phosphorylation of ERK induces DNA binding and trans-activation capacity of AP-1 and ETS, which are both crucial transcription factors of MMPs [10]. It is reported that activation of PI3K-AKT pathway can increase the expression of MMP-2 directly, phosphorylate IKKs and activates NF-κB pathway, which promotes NF-κB translocation to the nucleus and then regulates NF-κB-dependent MMP transcription [11]. Therefore, it is hopeful that chemical compounds targeting the Ras-mediated downstream pathways can be used as an inhibitor of MMP expression and therapeutic approaches of metastatic melanoma.

*Scutellaria baicalensis Georgi* is a medicinal herb widely used for the treatment of various inflammatory diseases, allergic reactions and cancer [12]. Wogonin (5,7–dihydroxy -8- methoxyflavone), one of the main active compounds of *scutellaria baicalensis*, has been considered to possess anticarcinogenic and chemopreventive abilities in a variety of experimental cancer models. The molecular target or the mechanism of action does not remain very clear. Recently, some reports indicated that anti-oxidant activity, anti-angiogenesis activity, the activation of impaired apoptosis and the activation of potential differentiation were the possible mechanisms of potent anticancer effect of wogonin. However, these multiple anti-cancer effects of wogonin could be related to regulate c-Myc/SKP2/Fbw7α and HDAC1/HDAC2 pathways [13]. It is also reported that wogonin had inhibitory effects on MMP-9 in MDA-MB-231 cells, which revealed anti-invasion potential [14]. Therefore, we would like to investigate the anti-invasion effect of wogonin on melanoma and discover the molecular target of wogonin.

In the present study, we used melanoma cells B16-F10 to investigate the inhibitory effects of wogonin on cell invasion and migration in *vitro* and in *vivo*, as well as the influences of wogonin on cytoskeleton and the expression and activity of MMP-2 and MMP-9. Furthermore, MAPK, AKT and NF-κB pathways, three key pathways mediating MMPs, were investigated to account for the molecular mechanism of this activity.

## Materials and Methods

### Ethics Statement

Animal study and euthanasia was carried out in strict accordance with the recommendations in the Guide for the Care and Use of Laboratory Animals of the National Institutes of Health. The protocol was approved by the Committee on the Ethics of Animal Experiments of the China Pharmaceutical University. The animals were maintained in a temperature and humidity controlled environment with a 12-h light and 12-h dark cycle. Feed and water were available *ad libitum*. All surgery was performed under sodium pentobarbital anesthesia and all efforts were made to minimize suffering.

### Materials

Wogonin was isolated from *scutellaria baicalensis* radix according to the method reported by Hui et al. [15]. Samples containing 99% or higher wogonin were used in the whole process of in *vitro* experiments. The compound was dissolved in dimethyl sulfoxide (DMSO) as a stock solution, stored at −20°C until needed. The final concentration of DMSO did not exceed 0.1% throughout the study (this concentration was found to have no effect on cell invasion or cell growth). Insulin-like growth factor-1 (IGF-1) and tumor necrosis factor-α (TNF-α) were obtained from PeproTech (Suzhou, China). Primary antibodies for MMP-2, MMP-9, ERK1/2, p-ERK1/2, PDK, IκBα, NF-κB (p65) and β-actin were purchased from Santa Cruz Biotechnology (Santa Cruz, CA), antibodies against Rac1, PI3K and Ras were from Bioworld (Bioworld, MN) and antibodies against AKT, p-AKT, p-IκBα (Ser32), IKKα, p-IKKα/β (Ser176/180) were from Cell Signaling Technology (Danvers, MA). MTT (3- (4, 5) -dimethylthiahiazo (-z-y1) -3, 5-diphenytetrazoliumromide) and fluorescein isothiocyanate (FITC)–phalloidin was from Sigma (St. Louis, MO). U0126 and LY294002 were from Beyotime (Shanghai, China). IRDyeTM800 conjugated second antibody was obtained from Rockland (Gilbertsville, PA).

### Animals and Cell culture

Male C57BL/6 mice (8–10 weeks old) weighting 20–25 g were obtained from the Shanghai Laboratory Animal Center (Shanghai, China). The highly metastatic melanoma cells B16-F10 were originally obtained from the Cell Bank of Shanghai Institute of Cell Biology. The cells were cultured in DMEM medium (Gibco, Grand Island, NY) containing 10% fetal bovine serum (Sijiqing, Hangzhou, China), 100 U/ml penicillin, and 100 mg/L streptomycin. All cell cultures were maintained at 37°C in a humidified atmosphere of 5% CO_2_.

### Cell viability assay

Cells were seeded at density of 10^4^ cells/ml and incubated with wogonin at various concentrations. After the exposure period, media was removed and cells were incubated with 20 µl 0.5% MTT in culture medium for an additional 4 h. The number of viable cells was directly proportional to the production of formazan, which was then solubilized with DMSO, and measured spectrophotometrically at 570 nm.

### Wound healing assay

B16-F10 motility was assessed using wound healing assay as described in a previous report [16]. Cells were seeded into a six-well plate for nearly 80% confluence. The confluent cell monolayers were wounded by a sterile white pipette tip. Then, the cell debris were washed away and replaced with 2 ml of fresh medium with different concentrations of wogonin for 24 h. The cells migrated into the cell-free space were measured using an inverted microscopy, five randomly chosen fields were analyzed for each well. Three independent experiments were performed.

### Cell attachment assay

The 96-well plates were coated with 5 mg/ml fibronectin (Sigma, St. Louis, MO) in PBS overnight at 4°C and blocked with 1% BSA for 4 h at 37°C. After cells were treated with different concentrations of wogonin for 24 h, cells were trypsinized and resuspended in serum-free DMEM medium at 5×10^5^ cells/ml. Aliquots (100 µl) of the cell suspensions were seeded into the wells and incubated for 1 h at 37°C. After that, unattached cells were washed thrice with PBS and the attached cells were determined by MTT assay [17]. The experiments were performed at least thrice.

### Cell invasion assay *in vitro*

Cell invasion assays were performed using transwell chamber (Corning Costar, Cambridge, MA). Matrigel (Becton Dickinson, Bedford, MA) was diluted to 5 mg/ml with DMEM serum-free medium, and applied to 8-µm pore size polycarbonate membrane filters of the chamber at 37°C for 1 h. The cells were seeded to the upper part of the chamber at a density of 5×10^5^ cells/ml in serum-free medium. In the lower chamber, DMEM medium containing 10% FBS served as a source of chemoattractants. After incubation, cells that had invaded through the matrigel and migrated to the bottom chamber were fixed in 100% methanol, stained with hematoxylin and eosin, photographed, and counted under a light microscope.

### Cell invasion assay *in vivo*

The melanoma cells B16-F10 were trypsinized and resuspended in culture media at a density of 1×10^6^ cells/ml and injected 0.2 ml into the tail veins of mice. Mice were weighed and randomly divided into five groups (each group contained ten mice): 0.9% normal saline control group, 100 mg/kg dacarbazine (DTIC), 15 mg/kg wogonin, 30 mg/kg wogonin and 60 mg/kg wogonin. The dosage of wogonin *in vivo* is determined by LD50 (half lethal dose) in nude mice and it is less than 1/6 of LD50. Test compounds were then administered daily injections for 20 days by intraperitoneal injection. Twenty-four hours after the last drug administration, the animals were sacrificed, the lungs were rapidly excised, washed, and fixed in Bouin's solution. The number of the tumor nodules on the whole surface of the lungs was counted under a dissecting microscope. Sections of each lung tissue sample were stained routinely with hematoxylin and eosin (HE) to confirm the formation of micrometastases.

### Western blotting

B16-F10 cells were treated with wogonin (15, 30 and 60 µM) for 24 h. IGF-1 (20 ng/ml) and TNF-α (20 ng/ml) were added respectively as the following described in figure legends. The cells were rinsed with PBS twice and were lysed in lysis buffer (50 mM Tris-Cl, pH 7.6, 150 mM NaCl, 1 mM EDTA, 1% (m/v) NP-40, 0.2 mM PMSF, 0.1 mM NaF and 1.0 mM DTT) on ice for 40 minutes. Cell lysate was then subjected to a centrifugation of 13,000×g for 10 min at 4°C to remove cell debris. Resultant protein samples were measured using BCA assay with a Varioskan multimode microplate spectrophotometer (Thermo, Waltham, MA). Equal quantities of protein were loaded onto SDS-polyacrylamide gels for separation and transferred onto nitrocellulose membranes by electroblotting. The blot was firstly incubated with 10% non-fat milk, followed by an 24 h incubation with the specific primary antibodies at 4°C. The final incubation was with IRDyeTM800 conjugated secondary antibody. Detection was carried out by the Odyssey Infrared Imaging System (LI-CORinc., Lincoln, MT).

### Gelatin zymography

After the B16-F10 cells were treated with 15, 30 and 60 µM wogonin in serum-free medium for 24 h, the supernatant was collected and mixed 3∶1 with loading buffer without heating or reduction to prepare samples for zymography analysis. The prepared samples were then subjected to electrophoresis on 10% SDS–PAGE containing 0.1% gelatin. After electrophoresis, the resulting gels were washed in 50 mM Tris-HCl (pH 7.6) containing 2.5% (v/v) Triton X-100 on a shaker for 30 minutes to remove SDS, and then incubated for 36 h in developing buffer (50 mM Tris–HCl, pH 7.6, 5 mM CaCl_2_, and 1 mM ZnCl_2_) at 37°C. The gel was stained with 0.1% Coomassie Brilliant Blue G250 for 1 h and destained in 10% acetic acid and 10% methanol.

### Preparation of cytosolic and nuclear extracts

After the treatment of wogonin (15, 30 and 60 µM) for 24 h, B16-F10 cells were harvested. Cells were lysed with buffer A (10 mM Hepes-KOH (pH 7.9), 10 mM KCl, 0.1 mM EDTA, 0.5% Nonidet P-40, 1 mM dithiothreitol, 0.5 mM phenylmethylsulfonyl fluoride), incubated on ice for 15 min to allow cells to swell and then centrifuged at 14,000 g for 15 min at 4°C. The supernatants were saved as the cytoplasmic fractions. The nuclear pellets were washed three times with buffer A and resuspended of the crude nuclei in high salt buffer (20 mM Hepes, 0.5 M KCl, 1 mM EDTA, 1 mM dithiothreitol, 1 mM phenylmethylsulfonyl fluoride, pH 7.9) for 30 min and then centrifuged at 12,000 rpm for 15 min at 4°C.

### Immunofluorescence

After the treatment of wogonin (0 and 60 µM) for 24 h, cells were fixed with 4% paraformaldehyde in PBS for 20 min, permeabilized with 0.5% Triton X-100 for 20 min, and blocked with 3% bovine serum albumin (BSA) for 1 h. FITC–phalloidin was used to probe the samples for 1 h to analyze actin remodeling.

### Statistical Analysis

The data shown in the study were obtained in at least five independent experiments and all results represented the mean ± S.E.M. Differences between the groups were assessed by one-way ANOVA and Dunnett's post hoc test. Comparisons were made relatively to the indicated groups, and the significance of differences was indicated as * P<0.05 and **P<0.01.

## Results

### Wogonin inhibits B16-F10 cells migration, adhesion and invasion *in vitro*

**Figure 1:**
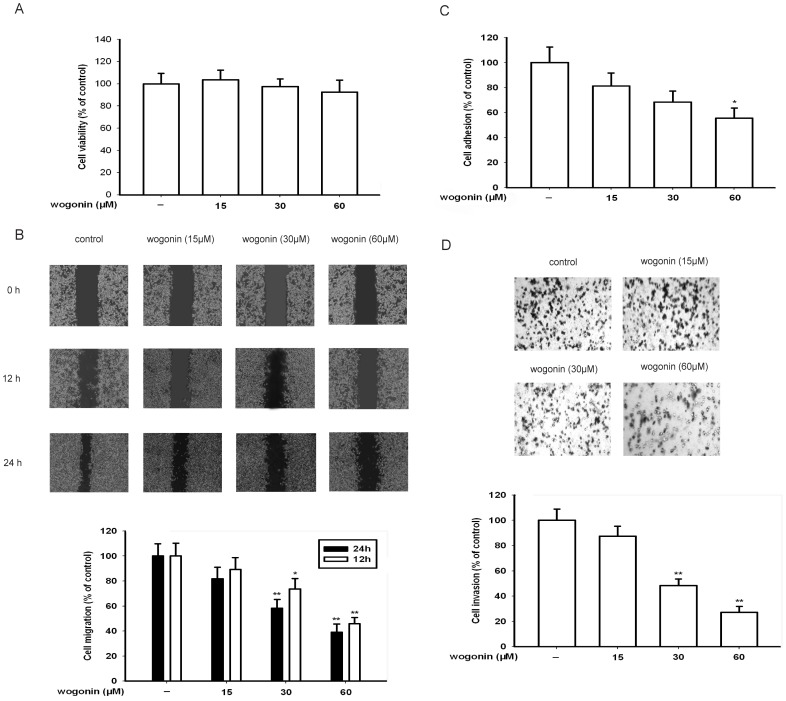
Effect of wogonin on B16-F10 melanoma cell viability, migration, adhesion and invasion in *vitro*. (A) Cells were exposed to different concentrations of wogonin for 24 h in 5% CO_2_ incubator at 37°C. Then, MTT assay was used to show wogonin has no effect on cell viability. (B) B16-F10 cells were scraped with a pipette tip and then treated with different concentrations of wogonin for 12 or 24 h. The migrating cells were assessed by microscope equipped with a camera. (C) After B16-F10 cells were treated with different concentrations of wogonin for 24 h, 100 µl cell suspension (5×10^5^ cells/ml) was then subjected to analyses for adhesion. The MTT assay shows that wogonin inhibits the cell adhesion. (D) After B16-F10 cells were treated with or without different concentrations of wogonin for 24 h, the invasive ability was evaluated by a matrigel-coated in *vitro* invasion assay. Each experiment was done at least three times. *p<0.05 compared with control; **p<0.01 compared with control.

As shown in Fig. 1A, a 24-h treatment of various concentrations (0–60 µM) of wogonin caused no detectable cytotoxicity which was measured by MTT assay (IC_50_ = 110 µM; data not shown). We next examined the effect of wogonin on the migration of B16-F10 cells grown in a six-well plate. As shown in Fig. 1B, the migrated cells were quantified by manual counting. When the percentage inhibition was expressed using untreated wells at 100%, the inhibition percentage of 15, 30 and 60 µM wogonin was about 19%, 42% and 64% respectively.

Cancer cell adhesion to basement membranes is important for cancer cell invasion since it can affect tumor cell locomotion and proteinase expression [18]. The results of cell attachment assay showed that the adhesive capabilities of B16-F10 were markedly decreased compared with the control after treatment of wogonin at 15, 30 and 60 µM for 24 h (Fig. 1C).

Then, we investigated the effects of wogonin on the invasion of B16-F10 melanoma cells in *vitro*. We found that cells treated with medium were able to migrate freely through the matrigel, whereas this ability was inhibited in cells treated with wogonin for 24 h. As shown in Fig. 1D, wogonin could inhibit the invasion of B16-F10 melanoma cells in a concentration-dependent manner, the inhibition percentage of 60 µM wogonin was about 75%.

### Wogonin inhibits B16-F10 cells invasion *in vivo*

**Figure 2:**
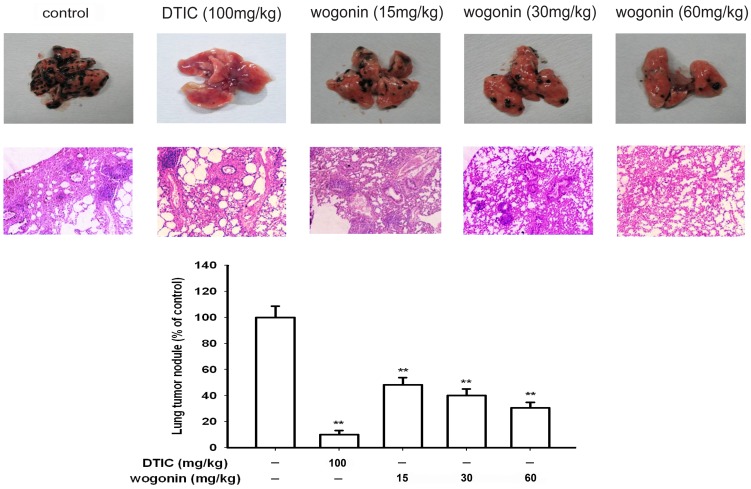
Effect of wogonin on B16-F10 melanoma cell invasion in *vivo*. Evaluation of macroscopically detect lung metastases. After fixation in Bouin's solution, the number of lung metastatic nodules on the surface was quantified. HE staining assay represent the metastases in the lungs of animals in each group. Each experiment was done at least three times. *p<0.05 compared with control; **p<0.01 compared with control.

To further confirm the effect of wogonin on the metastatic potential of the melanoma cells B16-F10 in *vivo*, the antimetastatic effect of wogonin was assessed in the C57BL/6 mice injected with B16-F10 cells. In this model study, we chose doses from 15 mg/kg to 60 mg/kg wogonin per day. As shown in Fig. 2A, there was a significant difference between the experimental groups and saline-treated group, the number of lung metastatic nodules in control group was 161.10±12.95, while only 82.30±8.37, 64.70±8.68 and 49.30±7.16 nodules were observed in the groups treated with wogonin at 15, 30 and 60 mg/kg, respectively. It was suggested that the metastatic effect of tumors was repressed by wogonin significantly in *vivo*. The HE staining assay was performed to reveal the histopathological changes of the lung tissue, which indicated that the metastatic nodules of control group were more and larger than that of DTIC or wogonin treated group (Fig. 2A).

### Wogonin inhibits MMP-2 and Rac1 protein expression in B16-F10 cells

**Figure 3:**
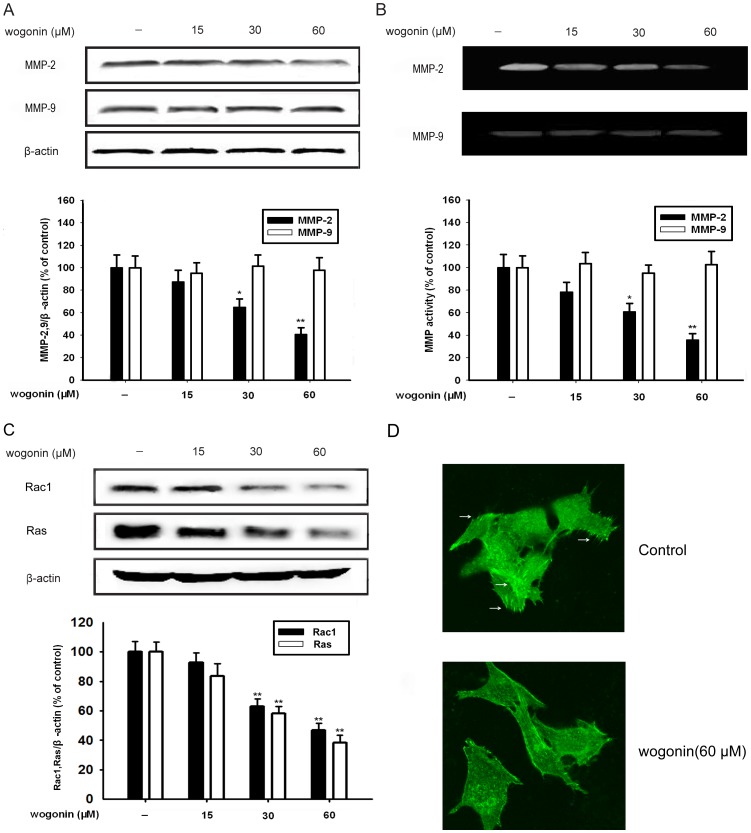
Effect of wogonin on MMP-2 and Rac1 protein expression in B16-F10 cells. B16-F10 cells were treated with indicated concentrations of wogonin for 24 h. (A) The expression of MMP-2 and MMP-9 protein in cells was analyzed by western blotting using specific antibodies. (B) The conditioned media was analyzed by the gelatin zymography to test the activity of MMP-2 and MMP-9. (C) The expression of Rac1 and Ras protein in cells was analyzed by western blotting using specific antibodies. An anti-β-actin antibody was used to check the proper protein loading. (D) F-actin fluorescence staining was performed to examine pseudopods formulation. Each experiments were done at least three times. *p<0.05 compared with control; **p<0.01 compared with control.

We carried out western blotting analysis to investigate whether wogonin inhibited the expression of MMP-2 and MMP-9 in B16-F10 cells under our experimental conditions. As shown in Fig. 3A, a concentration-dependent reduction of MMP-2 expression was detected in wogonin-treated B16-F10 cells. With the increment of wogonin concentration from 15 µM to 60 µM, the inhibition rate of MMP-2 increased from 16% to 60%, accordingly. However, wogonin did not have influence on MMP-9 expression.

In cell migration and invasion processes, ECM degradation is substantial, which suggests that matrix-degrading proteinases are required [19]. To clarify the involvement of wogonin in inhibiting the activity of MMP-2 and MMP-9, the B16-F10 cells cultured in conditioned medium was subjected to the gelatin zymography in the presence of various concentrations of wogonin. As shown in Fig. 3B, MMP-2 activity was significantly reduced by wogonin in a concentration-dependent manner. The inhibiting rate of MMP-2 reached to 67% when the concentration of wogonin was 60 µM. However, wogonin showed little effect on the activity of MMP-9 in B16-F10 cells when compared with the control group.

We also found wogonin (15, 30 and 60 µM) could inhibit the expression of small G protein Ras (by 17%, 42% and 62%) and Rac1 (by 8%, 38% and 54%) (Fig. 3B). Ras and Rac are both GTPases that function as molecular switches for various cellular activities. Rac1 can stimulate actin polymerization and regulate changes in cytoskeletal structure that affect cell shape, migration, and adhesion. Wogonin (60 µM) could reduce the number of pseudopodia formated by F-actin, thus inhibiting the migration of B16F10 cells.

### Wogonin inhibits the phosphorylation of ERK and AKT

**Figure 4:**
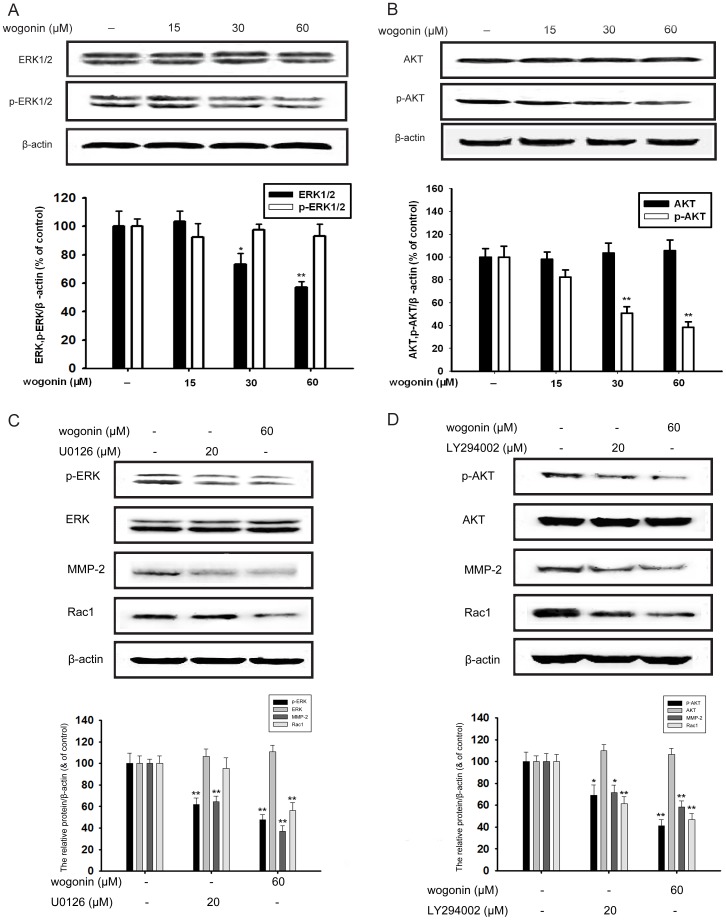
Effect of wogonin on the EKR and AKT signaling in B16-F10 cells. (A-B) B16-F10 cells were pretreated with different concentrations of wogonin for 24 h, and the cellular extracts were then blotted using specific antibodies. Phosphorylated protein expression of p-ERK and p-AKT was tested. Corresponding to the phosphorylation level, respective total amount of ERK1/2 and AKT were detected. (C) B16-F10 cells were pretreated with wogonin (60 µM) and U0126 (20 µM) for 24 h. The expression of ERK, p-ERK, MMP-2 and Rac1 protein in cells was analyzed by western blotting using specific antibodies. (D) B16-F10 cells were pretreated with wogonin (60 µM) and LY294002 (20 µM) for 24 h. The expression of AKT, p-AKT, MMP-2 and Rac1 protein in cells was analyzed by western blotting using specific antibodies. An anti-β-actin antibody was used to check the proper protein loading. Western blotting was done at least three times. *p<0.05 compared with control; **p<0.01 compared with control.

ERK and PI3K/AKT, which are both important Ras effectors in tumorigenesis, are involved in the regulation of MMP-2 [20]. We used western blotting assay to test the total and phosphorylation of ERK1/2 and AKT expression. As shown in Fig. 4D, wogonin inhibited the phosphorylation of ERK1/2 and AKT in a concentration-dependent manner without affecting the overall protein level of ERK1/2 and AKT, while the inhibition percentage of 60 µM wogonin was about and 43% and 64% (Fig. 4A and 4B).

Specific inhibitor U0126 against phosphorylated-ERK and LY294002 against phosphorylated-AKT was used to testify the inhibitory effect of ERK and AKT pathways, and then to observe the influence of wogonin on MMP-2 and Rac1. U0126 (20 µM) and wogonin (60 µM) could both inhibit the phosphorylation of ERK1/2 (38% and 51%, respectively) and there was little statistical differences in this inhibitory effect, while the inhibition of MMP-2 and Rac1 expression by wogonin (60 µM) is more obvious (63% and 44%, respectively) (Fig. 4C). In addition, ly294002 (20 µM) and wogonin (60 µM) could both inhibit the phosphorylation of ERK1/2 (30% and 60%, respectively) and wogonin showed a better inhibition on MMP-2 (52%) and Rac1(54%) expression (Fig. 4D).

### Wogonin inhibits AKT/PI3K and NF-κB pathways

**Figure 5:**
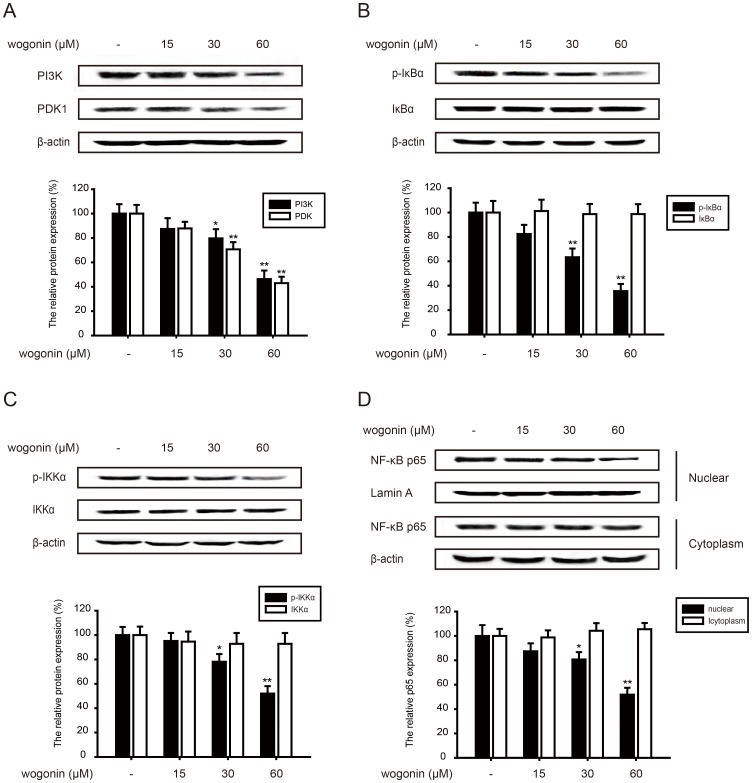
Effect of wogonin on AKT/PI3K and NF-κB pathways. B16-F10 cells were pre-treated with different concentrations of wogonin for 24 h. (A) Effects of wogonin on the protein levels of PI3K and PDK1 were examined. (B-C) The related proteins expression of NF-κB pathways (p-IKKα, IKKα, p-IκBα and IκBα) was tested. (D) Cytosolic fractions and nuclear extracts were prepared. Western blotting analyses was performed to investigate nuclear translocation of NF-κB p65. All protein was determined with specific antibodies. β-actin antibody was used to check the proper protein loading. Western blotting was done at least three times. *p<0.05 compared with control; **p<0.01 compared with control.

Wogonin showed the effect on the phosphorylation of AKT and further study had been focused on the upstream and downstream of AKT. AKT is phosphorylated and activated by the binding of its homology domain to phosphatidylinositol 3,4,5-trisphosphate (PIP3) and phosphatidylinositol 3,4-bisphosphate (PIP2), which are the main products of PI3K. This cascade signal transduction is also dependent on 3-phosphoinositide-dependent kinase 1(PDK1). Our results showed wogonin (15, 30 and 60 µM) down-regulated the protein expression of PI3K (13%, 21%, 54%) and PDK1 (12%, 30%, 58%), which indicated the inhibitory effect of AKT/PI3K pathway (Fig. 5A).

PI3K/AKT can phosphorylate IκB which is the endogenous inhibitors of NF-κB p65, thus releasing NF-κB p65 to cell nucleus. NF-κB is also the mediator of MMP-2, so the related protein expression of NF-κB pathways was investigated. Wogonin (15, 30 and 60 µM) suppressed the phosphorylation of IκBα (17%, 37%, 65%) (Fig. 5B) and IKKα (8%, 20%, 49%) without affecting the total protein level (Fig. 5C). Moreover, wogonin suppressed p65 nuclear expression while had almost no inhibition of the cytoplasmic p65 expression.

### Wogonin inhibits IGF-1-induced invasion through AKT/PI3K and NF-κB pathways

**Figure 6:**
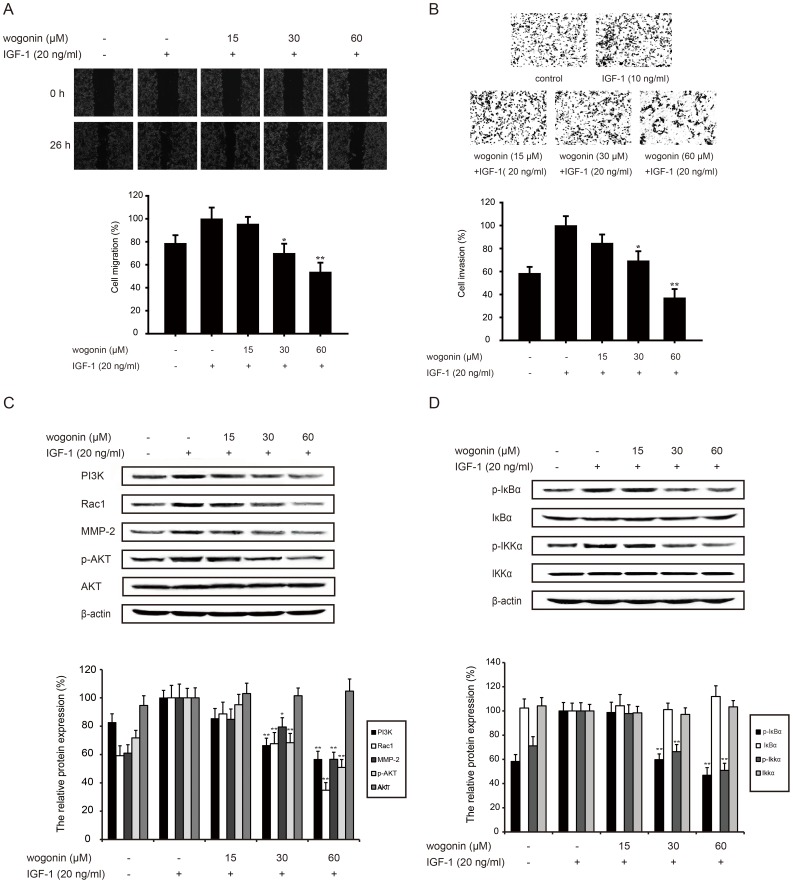
Wogonin inhibits IGF-1-induced invasion through AKT/PI3K and NF-κB pathways. B16-F10 cells were pretreated with different concentrations of wogonin with IGF-1 (20 ng/ml) for 24 h. (A) B16-F10 cells were scraped with a pipette tip. After 24 h-treatment, migration was assessed by microscope. (B) Pretreated cells were seeded in the upside of transwell, and the membrane was stained with hematoxylin and eosin after 24 h-incubation. (C) Western blotting analyses of the expression of MMP-2 and AKT/PI3K (PI3K, Rac1, p-AKT and AKT) pathway-related protein was performed. (D) NF-κB pathway-related protein (p-IKKα, IKKα, p-IκBα and IκBα) was determined by western blot assay. All protein was determined with specific antibodies. β-actin antibody was used to check the proper protein loading. Each experiment was done at least three times. *p<0.05 compared with IGF-1-treated group; **p<0.01 compared IGF-1-treated group.

To further prove the molecular mechanism, IGF-1 which acted as an activator of AKT/PI3K pathway was added together with wogonin for 24 h to test the inhibitory effect of wogonin. As shown in Fig. 6A and B, wogonin (15, 30 and 60 µM) could inhibit IGF-1-stimulated migrarion (5%, 30%, 47%) and invasion (15%, 31%, 63%) of B16-F10 melanoma cells in a concentration-dependent manner. The results also showed that wogonin (15, 30 and 60 µM) could inhibit expression of MMP-2 (15%, 21%, 44%) and Rac1 (11%, 32%, 65%) which were over-expressed after IGF-1 treatment (Fig. 6C).

We also found that wogonin (15, 30 and 60 µM) could down-regulat PI3K (16%, 34%, 43%) protein expression, and decreas p-AKT expression (5%, 32%, 49%) without affecting the total protein level (Fig. 6C).

It is well documented that NF-κB is the down-stream of AKT/PI3K pathways. NF-κB signaling was activated by IGF-1 following with the activation of AKT/PI3K pathways. Our results revealed that wogonin could inhibit NF-κB signaling with the 2 h-treatment of IGF-1. Wogonin (15, 30 and 60 µM) suppressed the expression of p-IκBα (2%, 41%, 54%) and p-IKKα (3%, 34%, 49%) induced by IGF-1(Fig. 6D). Yet, wogonin did not have any effect on the overall protein of IκBα and IKKα.

### Wogonin inhibits TNF-α-induced invasion through NF-κB pathway

**Figure 7:**
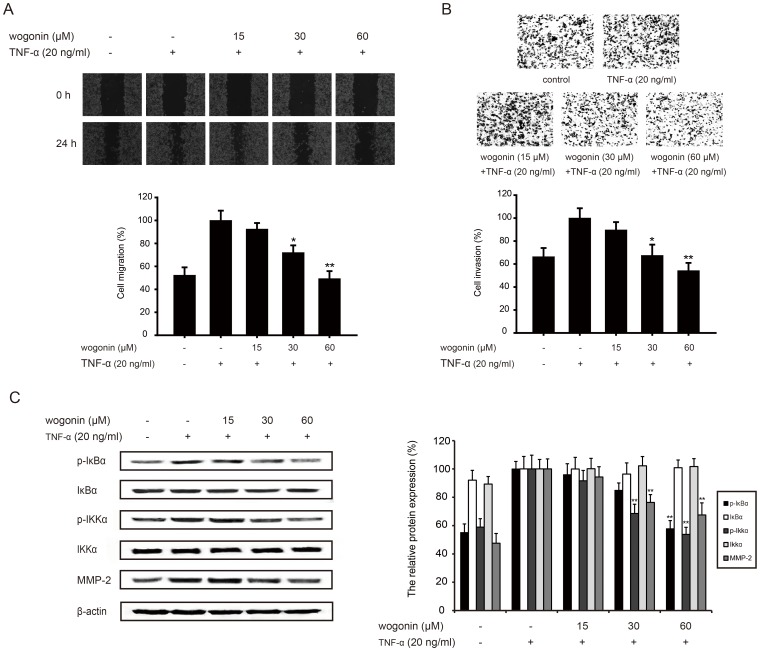
Wogonin inhibits TNF-α-induced invasion through NF-κB pathways. B16-F10 cells were pretreated with TNF-α (20 ng/ml) and different concentrations of wogonin for 24 h. (A) B16-F10 cells were scraped with a pipette tip before the treatment of wogonin and TNF-α. The strach was photographed by microscope. (B) Pretreated cells were counted and cultured in the upside of transwell coated with the matrigel, and the cells through the matrigel were stained with hematoxylin and eosin after 24 h-incubation. (C) Western blotting analyses of MMP-2 and NF-κB pathway-related protein (p-IKKα, IKKα, p-IκBα and IκBα) was performed. All protein was determined with specific antibodies. β-actin antibody was used to check the proper protein loading. Each experiment was done at least three times. *p<0.05 compared with TNF-α-treated group; **p<0.01 compared with TNF-α-treated group.

Since wogonin could inhibit NF-κB pathway, TNF-α was used as an inducer to further testify this effect. Wogonin (15, 30 and 60 µM) could inhibit TNF-α-stimulated B16-F10 cells from acrossing the wounded space (Fig. 7A) and decrease the more invasive cells through the matrigel (Fig. 7B) in a concentration-dependent manner.

Our results also showed wogonin together with 24-h treatment of TNF-α(15, 30 and 60 µM) suppressed the phosphorylation of IκBα (4%, 15%, 43%) and IKKα (8%, 32%, 47%) (Fig. 7C), but they had almost no influence on the total protein level of IκBα and IKKα. Wogonin blocked TNF-α-activated NF-κB pathway and down-regulated MMP-2 expression (6%, 24%, 33%), thus inhibiting TNF-α-induced invasion.

## Discussion

Metastasis of tumor is the most important cause of mortality worldwide [21]. Melanoma cells invade and metastasize during the early stage of tumorigenesis [22]. Tumor invasion and metastasis are complex processes which occur by a series of complex events including cell migration, adhesion and invasion. In this study, according to wound healing, adhesion and invasion assays, wogonin effectively inhibited the migration and invasion of B16-F10 cells in *vitro*. Meanwhile, wogonin could repress the metastatic of B16-F10 melanoma cells in *vivo* by the tail veins of C57BL/6 mice experiment. Our studies suggested that wogonin effectively inhibited the metastasis, invasion, and migration of highly metastatic B16-F10 melanoma cells and promised to be a potential drug for metastasic melanoma.

MMPs, a family of endopeptidases, is considered to be one of the main intracellular factors responsible for the tumor invasion and tissue remodeling [23]. Our present results had demonstrated that wogonin suppressed the expression and activity of MMP-2 in B16-F10 cells, which indicated its anti-invasion activity, while our previous study indicated that wogonin possessed an inhibitory effect on the expression and activity on MMP-9 in MDA-MB-231 cells. MMP-2 is the most important member of the MMPs family. Membrane-type MMPs combine TIMP-2 as a complex and then activate MMP-2. The activity of MMP-9 is medicated by MMP-2 and many other proteases such as MMP-1 and MMP-3 [24]. Protease network at the cellular surface is complicated and the function of MMPs varies in different tumors. MMP-2 is a biomolecular and indicates prognosis in melanoma [6]. Most patients who have melanoma die of hematogenous and lymphatic metastasis. MMP-2 can not only degrade physical barriers like ECM, but also function as a key factor in angiogenic or lymphangioenic processes [25]. As MMP-2 plays multiple roles in tumor in *vivo*, wogonin could inhibit the metastasis process through the suppression of MMP-2 expression and activity. In addition, wogonin inhibited the expression of Rac1, which is a Ras-related protein that regulate cytoskeletal structure and mediate cellular signaling. It indicated that wogonin could inhibit migration and comprehensively regulate signal transduction.

For many tumor harbors *ras* gene mutations, Ras proteins have become the focus of research in cancer drug discovery [26]. Although malignant melanomas mainly have activating mutations in N-Ras and wogonin inhibited Ras protein expression, wogonin's effect on mutant forms of Ras proteins remains unclear and tumors are usually controlled by a single oncogene. The current opinion of drug discovery strategy is to target Ras downstream signaling pathways, such as ERK or AKT pathways, and many drugs targeting Ras effectors remain in early phase trials. Interestingly, there have been reports showing that MMP-2, which is considered to be a therapeutic target of highly metastatic cells, is an ERK and PI3K-regulated gene and is associated with invasion and metastasis. Therefore, we investigated the effect of wogonin on the total ERK1/2 and AKT as well as their phosphorylated forms in B16-F10 melanoma cells. Wogonin has been shown to suppress the phosphorylation of ERK and AKT while wogonin showed a more obvious inhibitory effect on MMP-2 and Rac1 expression than the specific inhibitor of ERK and AKT. It indicated that blockage of ERK and AKT pathways together is more effective in suppressing melanoma invasion and migration. In addition, wogonin could also suppress the expression of PI3K and PDK1. Therefore, we conclude that the suppression of phosphorylation of AKT is through PI3K/AKT signaling and then wogonin could possibly inhibit the related downstream targets such as MMP-2. Moreover, wogonin inhibited the phosphorylation of IKKα and IκBα and nuclear localization of NF-κB p65, which suggested a blockage in NF-κB pathway. NF-κB is one of the important factors that regulate the transcriptional activity MMP-2. A number of reports have convincingly demonstrated that the blockage in NF-κB pathway leads to suppress the expression of MMP-2 and MMP-9. Interestingly, it is known that PI3K/AKT pathway activates the NF-κB system. Our results showed that wogonin inhibited both PI3K/AKT and NF-κB pathway. However, we did not investigate that the inhibition of NF-κB pathway was dependent on PI3K/AKT pathway. Studies need to be done to further confirm that whether wogonin inhibited NF-κB pathway directly through PI3K/AKT pathway or indirectly.

It is documented that IGF-1 plays roles in initial steps of malignant transformation and multiple steps of metastasis [27]. TNF-α is also frequently found overexpressing in many human cancer tissue [28]. IGF-1 and TNF-α can activate tumor microenvironment and aid tumor cells in escaping from the primary tumor. IGF-1 and TNF-α bind to their receptors, which leads to promoting a recruitment of adaptor proteins and activating signal cascades of PI3K/AKT and NF-κB pathway. To further confirm the roles of PI3K/AKT and NF-κB pathway in inhibition of MMP-2 by wogonin, IGF-1 was used as an activator of PI3K/AKT pathway and TNF-α as that of NF-κB pathway. The results revealed that wogonin could inhibit invasion of B16-F10 cells and suppress PI3K/AKT and NF-κB pathway after the stimulation of IGF-1. NF-κB pathway was also inhibited by wogonin with the treatment of TNF-α, thus decreasing the expression of MMP-2. In consequence, wogonin could down-regulate the expression of MMP-2 through PI3K/AKT and NF-κB pathway. In addition, wogonin could inhibit IGF-1-activated PI3K/AKT/NF-κB signaling. However, whether wogonin can inhibit NF-κB signaling without the function of AKT exists to be researched. On the other hand, both IGF-1 and TNF-α are involved in the formation of reactive tumor microenvironment. This reactive tumor microenvironment can enhance the invasion and migration of cancer cells. As a result, wogonin inhibited invasion and migration induced by IGF-1 or TNF-α, which further showed its anti-invasion and anti-migration potential.

Although B16-F10 melanoma cells are derived from mouse, our research adds new evidence of wogonin against melanoma and underlies our further research on human melanoma A375 cells. We found that wogonin not only inhibited the migration and invasion in A375 cells and TNF-α-induced A375 cells, but also surpressed the Ras expression and Ras-medicated ERK and AKT pathways (Fig. S1 and S2). Interestingly, our former study has demonstrated that wogonin inhibited the invasion in KRAS and BRAF-mutated MDA-MB-231 through ERK pathway. In this study, we further found that wogonin could inhibit the expression of Ras and block AKT pathway, which showed the potential molecular target of wogonin (Fig. S2).

In conclusion, we demonstrated that wogonin could inhibit the invasion and metastasis of B16-F10 melanoma cells in *vitro* and in *vivo*. By studying the molecular mechanisms of wogonin against B16-F10 melanoma cells, we have confirmed that wogonin inhibited Ras expression, as well as ERK, AKT and NF-κB pathways, which are the possible upstream targets of MMP-2. Taken together with previous reports about its antitumor effect, wogonin appears to be a promising therapeutic drug of melanoma.

## Supporting Information

Figure S1 **Wogonin inhibits motility and invasion in human melanoma A375 cells.** (A) A375 cells were scraped with a pipette tip and then treated with different concentrations of wogonin for 24 h. (B) A375 cells were scraped with a pipette tip and then treated with TNF-α (20 ng/ml) and different concentrations of wogonin for 24 h. Migrated cells were assessed by microscope equipped with a camera. (C-D) Pretreated cells were counted and cultured in the upside of transwell coated with the matrigel, and the cells through the matrigel were stained with hematoxylin and eosin after 24 h-incubation. Each experiment was done at least three times. *p<0.05 compared with the control or TNF-α-treated group; **p<0.01 compared with the control or TNF-α-treated group.
(TIF)

Figure S2 **Effects of wogonin on Ras expression, ERK and AKT pathways in A375 and MDA-MB-231 cells.** (A) A375 cells were pretreated with different concentrations of wogonin for 24 h, and the cellular extracts were then blotted using specific antibodies. The protein expression of Ras, p-ERK, p-AKT PI3K and respective total amount of ERK and AKT were detected. (B) MDA-MB-231 cells were pretreated with different concentrations of wogonin for 24 h. The protein expression of Ras, p-AKT, PI3K and respective total amount AKT were detected by western blot experiment using specific antibodies. An anti-β-actin antibody was used to check the proper protein loading. Western blotting was done at least three times. *p<0.05 compared with control; **p<0.01 compared with control.
(TIF)
