# Supplementary figures and images for: Wogonin Suppresses Melanoma Cell B16-F10 Invasion and Migration by Inhibiting Ras-Medicated Pathways
Source: PLoS One. 2014 Sep 9;9(9):e106458. doi: 10.1371/journal.pone.0106458 (PMC4159230; doi:10.1371/journal.pone.0106458)

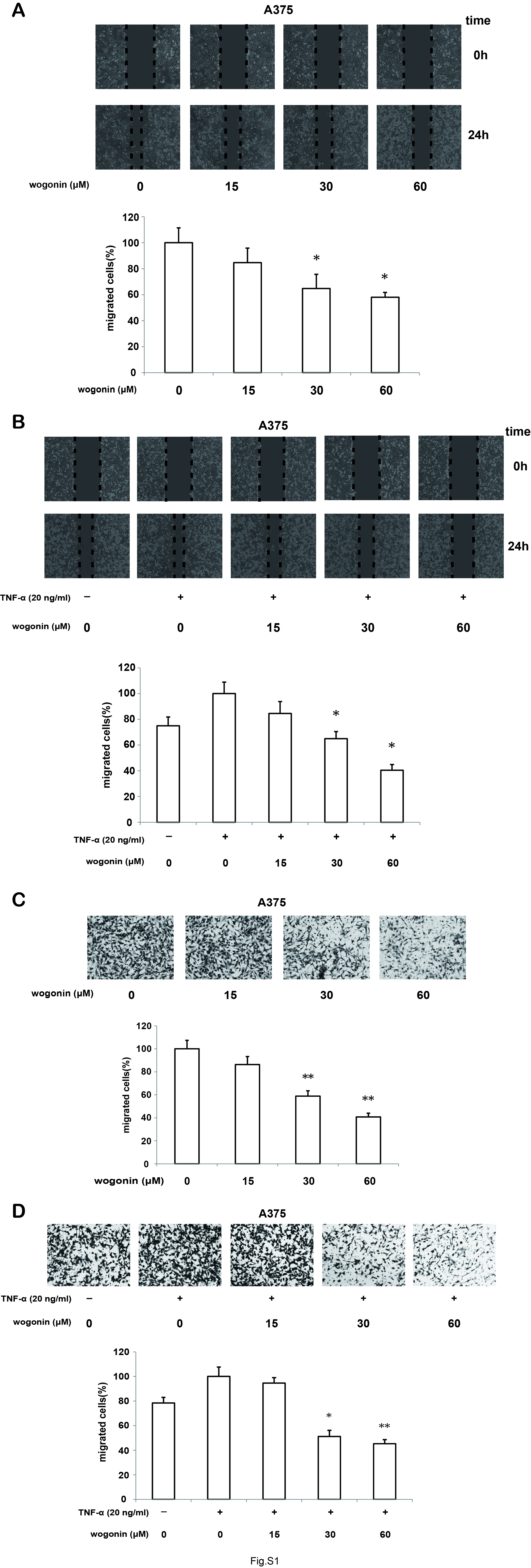

Supplement: Figure S1 — Wogonin inhibits motility and invasion in human melanoma A375 cells. (A) A375 cells were scraped with a pipette tip and then treated with different concentrations of wogonin for 24 h. (B) A375 cells were scraped with a pipette tip and then treated with TNF-α (20 ng/ml) and different concentrations of wogonin for 24 h. Migrated cells were assessed by microscope equipped with a camera. (C-D) Pretreated cells were counted and cultured in the upside of transwell coated with the matrigel, and the cells through the matrigel were stained with hematoxylin and eosin after 24 h-incubation. Each experiment was done at least three times. *p<0.05 compared with the control or TNF-α-treated group; **p<0.01 compared with the control or TNF-α-treated group. (TIF) [file pone.0106458.s001.tif]

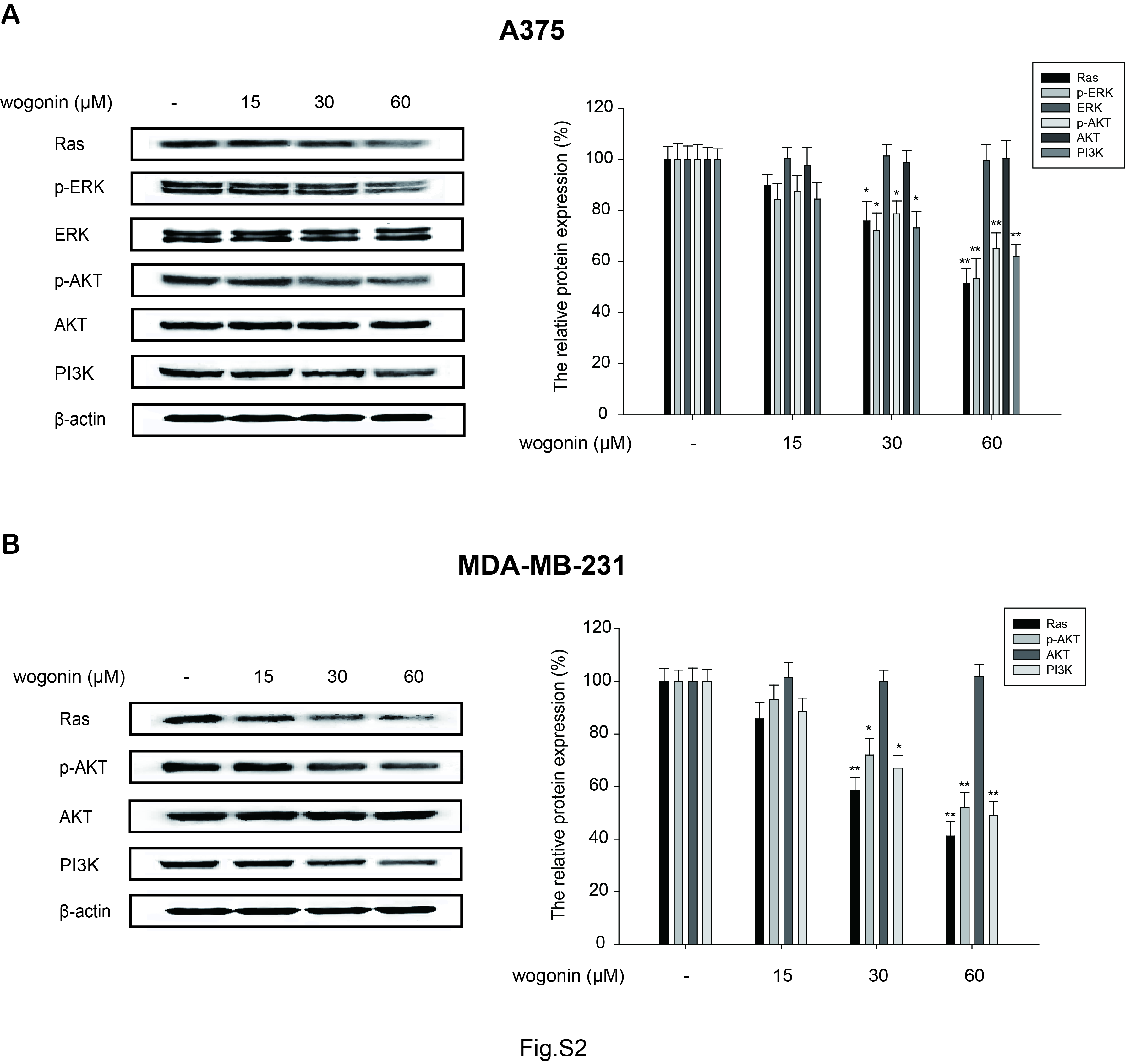

Supplement: Figure S2 — Effects of wogonin on Ras expression, ERK and AKT pathways in A375 and MDA-MB-231 cells. (A) A375 cells were pretreated with different concentrations of wogonin for 24 h, and the cellular extracts were then blotted using specific antibodies. The protein expression of Ras, p-ERK, p-AKT PI3K and respective total amount of ERK and AKT were detected. (B) MDA-MB-231 cells were pretreated with different concentrations of wogonin for 24 h. The protein expression of Ras, p-AKT, PI3K and respective total amount AKT were detected by western blot experiment using specific antibodies. An anti-β-actin antibody was used to check the proper protein loading. Western blotting was done at least three times. *p<0.05 compared with control; **p<0.01 compared with control. (TIF) [file pone.0106458.s002.tif]
